# A tubulin-binding protein that preferentially binds to GDP-tubulin and promotes GTP exchange

**DOI:** 10.1016/j.jbc.2025.110401

**Published:** 2025-06-19

**Authors:** Wesley J. Yon, Taekjip Ha, Yixian Zheng, Ross T.A. Pedersen

**Affiliations:** 1 Department of Embryology, Carnegie Institution for Science, Baltimore, Maryland, USA; 2 Cell, Molecular, Developmental Biology, and Biophysics Program, Johns Hopkins University, Baltimore, Maryland, USA; 3 Department of Pediatrics, Harvard Medical School, Boston, Massachusetts, USA; 4 Howard Hughes Medical Institute and Program in Cellular and Molecular Medicine, Boston Children's Hospital, Boston, Massachusetts, USA

**Keywords:** GTPase, guanine nucleotide exchange factor (GEF), microtubule, microtubule-associated protein (MAP), tubulin

## Abstract

**α**- and **β**-tubulin form GTPase heterodimers and assemble into microtubules. Like other GTPases, the tubulin heterodimer's nucleotide-bound state regulates its activity. In the dimer, **α**-tubulin is constitutively bound to GTP, while **β**-tubulin can bind to either GDP (GDP-tubulin) or GTP (GTP-tubulin). Following assembly into microtubules, GTP-tubulin hydrolyzes GTP to GDP, triggering microtubule disassembly. This generates free GDP-tubulin, which must exchange GDP for GTP to undergo assembly again. Tubulin dimers undergo rapid nucleotide exchange *in vitro*, leading to a commonly accepted belief that a tubulin guanine nucleotide exchange factor (GEF) may be unnecessary for microtubule assembly in cells. Here, we use quantitative binding assays to show that BuGZ, a spindle assembly factor, binds tightly to GDP-tubulin, less tightly to GTP-tubulin, and weakly to microtubules. We further show that BuGZ promotes the incorporation of GTP into tubulin using a nucleotide exchange assay. The discovery of a tubulin GEF suggests a mechanism that may aid rapid microtubule assembly dynamics in cells.

Microtubules are one eukaryotic cytoskeletal element required for key cellular functions including transport of cargos *via* motor proteins, the relay of mechanical signals to the interphase nucleus, and proper segregation of chromosomes during cell division (1, 2, 3, 4).

Microtubules are assembled from tubulin dimers, which comprise one α- and one β-tubulin protein. In a dimer, α-tubulin is constitutively bound to guanosine triphosphate (GTP) at its non-hydrolyzing, non-exchangeable “N-site.” β-tubulin binds to and rapidly exchanges GTP or GDP at its exchangeable “E-site,” where GTP can also be hydrolyzed into GDP (5, 6). Tubulin heterodimers with GTP-bound β-tubulin (hereinafter referred to as GTP-tubulin) polymerize into microtubules (7). Microtubules grow by binding GTP-tubulin dimers at one end. As the microtubule grows, longitudinal and lateral interactions of incorporated dimers activate β-tubulin GTPase activity, stochastically hydrolyzing the bound GTP and forming GDP-tubulin in the microtubule lattice (8, 9). As a result, the growing ends of microtubules contain GTP-tubulin, called the GTP cap. GTP hydrolysis induces tubulin dimer conformational changes which cause destabilization of the microtubule (10). In the absence of the GTP cap, microtubules transition from growth to shrinkage, termed catastrophe (11). Microtubule disassembly releases free GDP-tubulin, which must exchange its bound GDP for GTP before it can polymerize again.

GTPases generally exhibit slow intrinsic rates of nucleotide exchange (t_1/2_ > 30 min) and require guanine nucleotide exchange factors (GEFs) to accelerate nucleotide dissociation by orders of magnitude for proper biological function (12, 13, 14, 15, 16, 17). GEFs have been characterized for myriad GTPase families, and GEFs within a family share common catalytic domains such as the CDC25 domain for RasGEFs or the Sec7 domain for ArfGEFs (18). GEFs between different GTPase families do not share homologous amino acid sequences, making it difficult to identify novel GEFs from primary structure alone (19). Despite the lack of sequence similarity, studies have uncovered a recurring GEF mechanism in which GEFs destabilize Mg^2+^ in the GTPase nucleotide binding pocket, which is required for stable nucleotide binding (20, 21). GEFs bind to GTPases without specificity to their nucleotide states and mediate the exchange of either bound GTP or GDP with free nucleotides in solution (12, 18, 22). In cells, the 10:1 ratio of intracellular GTP:GDP ensures that the GTP-bound form is the dominant GTPase species when a GEF is present (23).

In contrast to the slow, measured rates of nucleotide exchange of many GTPases, the *in vitro* dissociation of GDP from β-tubulin is rapid (t_1/2_ ≅ 5 s) (6). Therefore, it is believed that free tubulin in cells quickly exchanges into the GTP-bound form, leading to the view that a tubulin GEF is not needed. However, microtubule assembly and disassembly dynamics increase dramatically in certain cellular processes, leading to rapid and continuous microtubule turnover. For example, microtubule turnover increases 18-fold in mitosis compared to interphase, a considerable increase in demand for GTP-tubulin (24). Additionally, microtubule dynamics are diffusion-limited in metaphase *Xenopus* egg extracts, and energy consumption outpaces energy production during cell division (25, 26). In this context, the more rapid dynamics of microtubules may necessitate a tubulin GEF.

BuGZ (Bub3-interacting and GLEBS motif-containing protein ZNF207) binds to tubulin and microtubules (27). In mitosis, BuGZ regulates spindle assembly by promoting microtubule polymerization and proper microtubule-kinetochore attachment by binding and stabilizing Bub3, a spindle assembly checkpoint protein (28, 29, 30). As a result, reduction of BuGZ results in prometaphase arrest with defects in spindle morphology and chromosome alignment (29, 30). BuGZ is conserved in animals and plants, and plant BuGZ may similarly regulate microtubule-kinetochore interactions (31, 32, 33). BuGZ's N-terminus, containing two zinc fingers, binds to tubulin, the mitotic kinase Aurora A, and microtubules. BuGZ undergoes liquid-liquid phase separation through its intrinsically disordered C-terminal region (27, 29, 34). In combination, the respective N- and C-terminal domains function cooperatively to concentrate tubulin and Aurora A, which in turn promote microtubule polymerization and Aurora A kinase activity, respectively (27, 34). The multivalent binding of phase-separated BuGZ also leads to microtubule bundling (27). Following the report of BuGZ promoting microtubule polymerization by concentrating tubulin within condensates, studies have identified similar behaviors in other phase-separating microtubule regulators, such as TPX2 and centrosome components, indicating that phase separation may play a role in mitotic spindle morphogenesis (35, 36).

Through quantitatively analyzing BuGZ's binding affinity to tubulin and microtubules, we found that BuGZ binds 10-fold more tightly to GDP-tubulin than to GTP-tubulin, and 210-fold more tightly to GDP-tubulin than to microtubules. We further present evidence that BuGZ promotes tubulin nucleotide exchange, converting GDP-tubulin to GTP-tubulin. We will discuss the implication of our findings on microtubule assembly dynamics.

## Results

### BuGZ binds weakly to microtubules

BuGZ binds to microtubules and tubulin dimers such that, when BuGZ phase separates, tubulin concentrates within the resulting BuGZ condensates, promoting microtubule polymerization (27, 29). BuGZ droplets formed along microtubules also bundle microtubules due to their multivalent microtubule interactions (27). However, BuGZ interactions with tubulin and microtubules are incompletely understood due to a lack of quantitative binding data. We performed quantitative assays to determine the binding affinity between *Xenopus laevis* BuGZ and microtubules.

**Figure 1:**
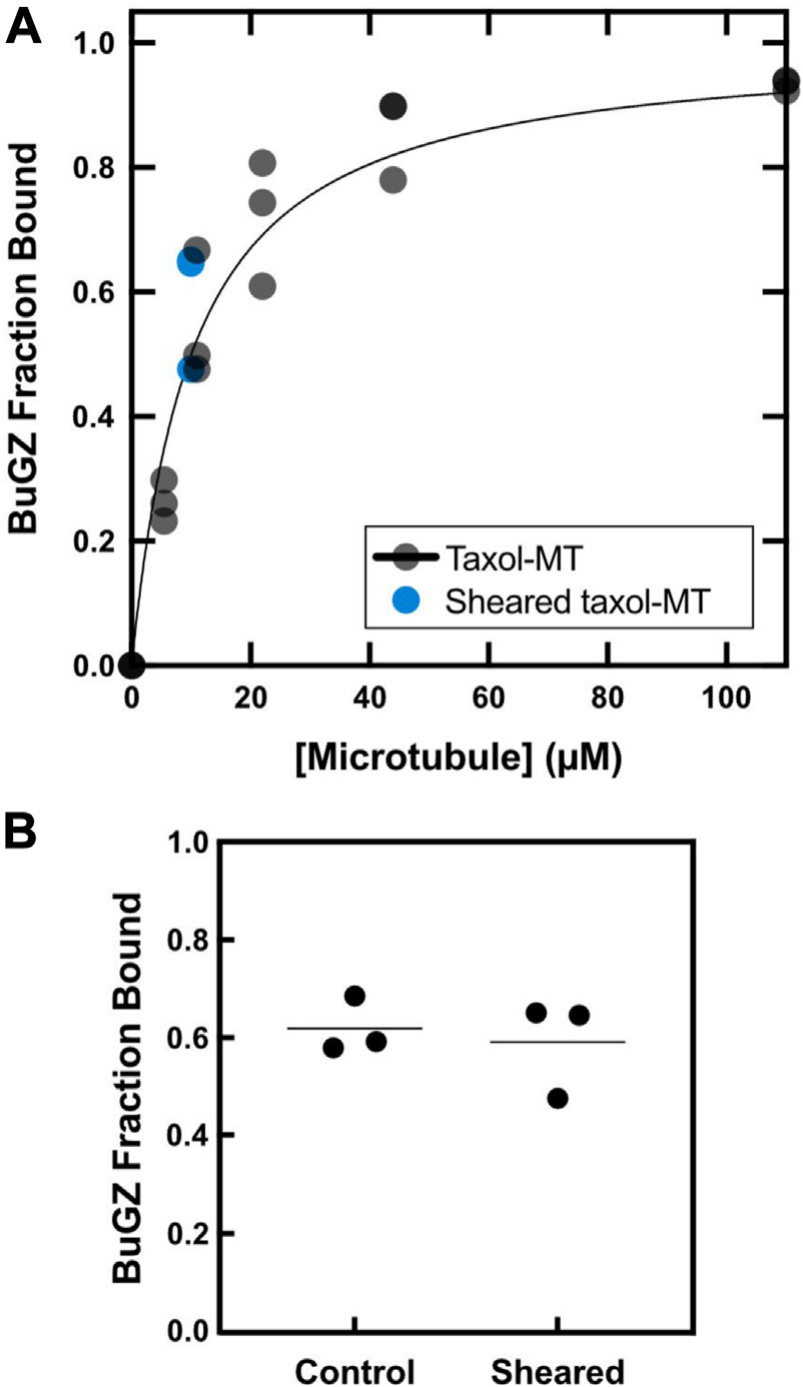
**BuGZ binds weakly to microtubules**. *A*, BuGZ binding isotherm to taxol-microtubules. *Black* data points are the BuGZ fraction bound. Curve-fit, Equation S1. Data from (*B*) for the BuGZ fraction bound to 10 μM sheared taxol-microtubules are shown in *blue*. *B*, BuGZ fraction bound to 10 μM taxol-microtubules and sheared taxol-microtubules. Data shown with the mean of three replicates.

To measure equilibrium binding of BuGZ to microtubules, we used taxol-stabilized microtubules polymerized with a 1:10 mixture of biotin-labeled: unlabeled GTP-tubulin such that ≥1 biotin-tubulin was present per 12-protofilament turn (37), facilitating retrieval of the microtubules with magnetic streptavidin beads. Since taxol-stabilized microtubules remain intact at low temperatures (Fig. S1, *A* and *B*), we performed all equilibrium binding experiments at 4 °C to suppress BuGZ phase separation (27, 38), which would confound analysis of concentration-dependent binding data. Under these conditions, we mixed varying concentrations of taxol-stabilized microtubules with streptavidin-coated magnetic beads. Purified BuGZ (Fig. S2*A*) was added to 1.19 μM, and the reaction was allowed to reach equilibrium. Then, microtubules and any BuGZ bound to them were retrieved with a magnet. The supernatant was collected, and BuGZ depletion was measured by quantitative immunoblotting. The fraction of BuGZ bound to microtubules was plotted against microtubule concentrations and then fitted with a rectangular hyperbola (Equation S1), and the equilibrium dissociation constant (K_D_) was determined from the fit. The K_D_ of BuGZ for taxol-stabilized microtubules is 9.45 μM (95% CI: 7.72 μM–11.50 μM) (Figs. 1*A*, S3*A*). A similar fraction of BuGZ was bound to 10 μM unsheared or sheared microtubules, demonstrating that BuGZ does not show an apparent preference for microtubule ends or lattice in our assay (Fig. 1, *A* and *B*).

### BuGZ preferentially binds GDP-tubulin over GTP-tubulin

**Figure 2:**
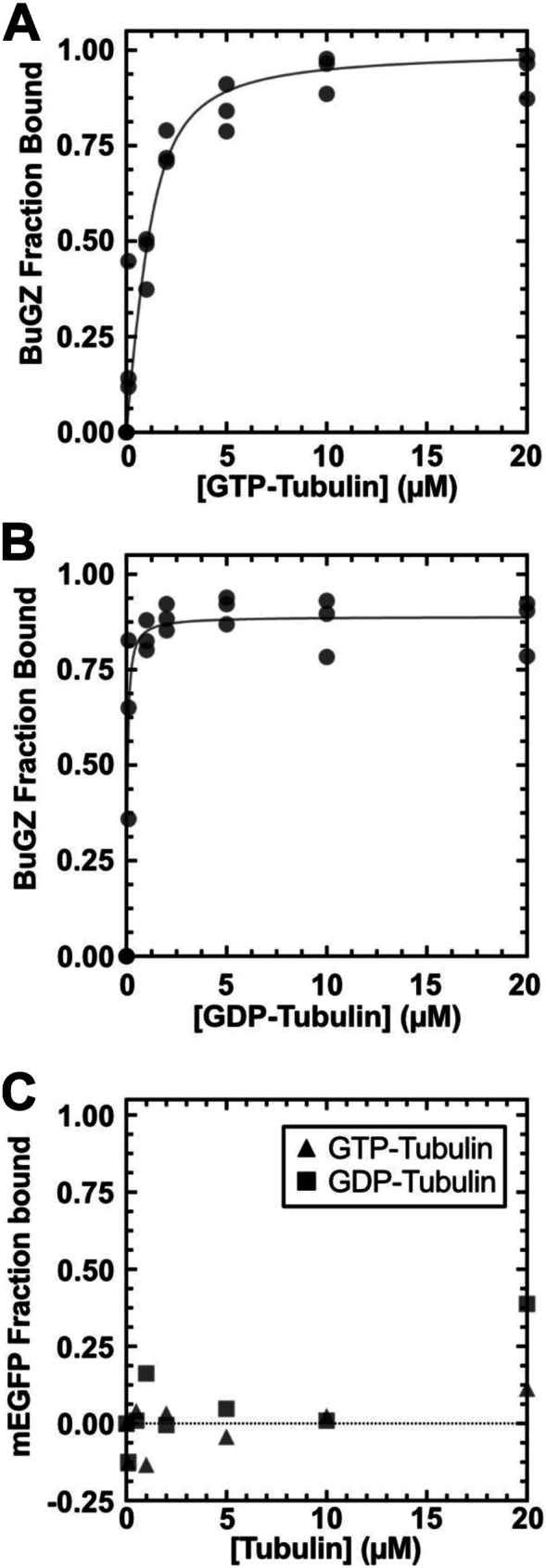
**BuGZ binds preferentially to GDP-tubulin over GTP-tubulin.***A*, BuGZ binding isotherm to GTP-tubulin. Curve-fit, Equation S1. *B*, BuGZ binding isotherm to GDP-tubulin. Curve-fit, Equation S2. *C*, mEGFP as a binding assay control binds similarly negligibly to GDP-tubulin and GTP-tubulin. Triangles, GTP-tubulin. Squares, GDP-tubulin.

The findings above show that the BuGZ binds microtubules weakly relative to other characterized microtubule-associated proteins (39, 40, 41, 42). Since BuGZ also binds to tubulin and BuGZ condensates concentrate tubulin dimers, we next measured how tightly BuGZ binds free tubulin dimers (27). We first investigated BuGZ's affinity for GTP-tubulin. Streptavidin-coated magnetic beads were incubated with varying concentrations of biotinylated GTP-tubulin and then BuGZ was added to a final concentration of 1.19 μM. The reaction was performed at 4 °C and in the presence of nocodazole to prevent the assembly of microtubules and minimize BuGZ phase separation. The beads were retrieved *via* magnetic separation, and BuGZ equilibrium binding was assessed by measuring BuGZ depletion from the supernatant *via* immunoblotting. The K_D_ for BuGZ binding to GTP-tubulin is 477 nM (95% CI: 275.1 nM–757.5 nM) (Figs. 2*A*, S3*B*). The 20-fold stronger binding affinity of BuGZ to GTP-tubulin than to microtubules prompted us to measure the binding affinity of BuGZ to GDP-tubulin using the same assay. The K_D_ of BuGZ for GDP-tubulin is 45.3 nM (95% CI: 21.7 nM–78.5 nM), 10-fold stronger than its affinity for GTP-tubulin and 210-fold stronger than for taxol-microtubules (Figs. 2*B*, S3*C*). Control experiments using mEGFP in solution with bead-bound tubulin showed no differential effect in GDP or GTP conditions (Fig. 2*C*).

### BuGZ’s N-terminus recognizes tubulin nucleotide state and phase separation increases tubulin binding affinity

**Figure 3:**
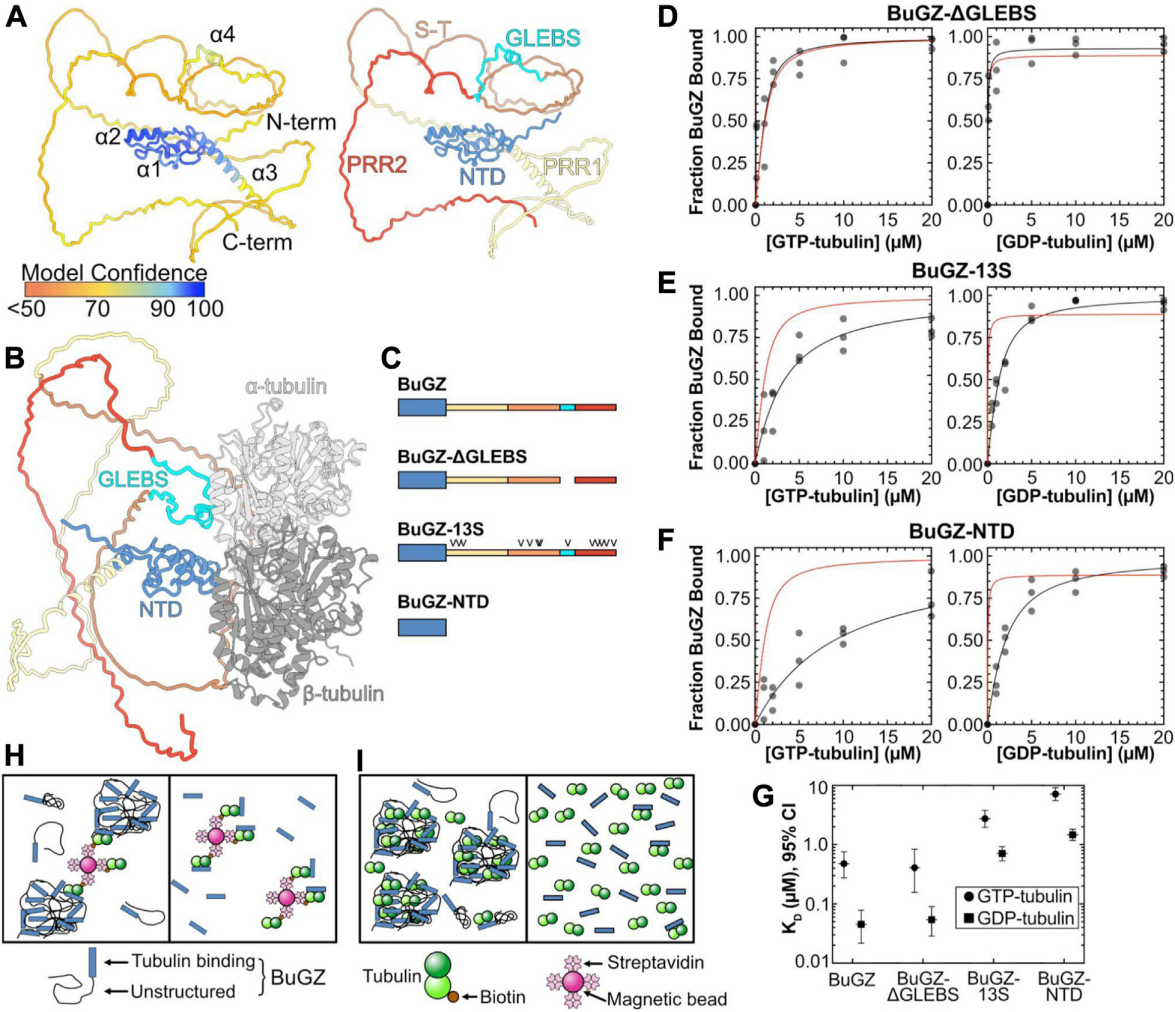
**BuGZ's N-terminal domain exhibits nucleotide-dependent binding to tubulin, but optimal BuGZ phase separation is required for tight binding.***A*, AlphaFold-predicted structure of *X. laevis* BuGZ. *Left*: color-coded by Model Confidence score. *Dark blue*, Very *high* (>90). *Light blue*, *High* (90–70). *Yellow*, Low (70–50). *Orange*, Very *low* (<50). *Right*: Color-coded by protein region. *Dark blue*, N-terminal domain (NTD). *Yellow*, proline-rich region 1 (PRR1). *Orange*, serine-threonine rich region (S-T). *Cyan*, GLEBS domain (GLEBS). *Red*, proline-rich region 2 (PRR2). *B*, AlphaFold-predicted structure of BuGZ bound to α-tubulin and β-tubulin. BuGZ color-coding is the same as (*A*, *right*). α-tubulin, *light gray*. β-tubulin, *dark gray*. *C*, schematics of BuGZ constructs. Domain color-coding is the same as (*A*, *right*). Chevrons indicate Ser substitutions in BuGZ-13S. *D*, BuGZ-ΔGLEBS binding isotherm to GTP-tubulin (*left*) and GDP-tubulin (*right*). *E*, BuGZ-13S binding isotherm to GTP-tubulin (*left*) and GDP-tubulin (*right*). (*F*) BuGZ-NTD binding isotherm to GTP-tubulin (*left*) and GDP-tubulin (*right*). Note: *Red lines* in *panels* (*D*, *E*, and *F*) show the binding curve of full-length wild-type BuGZ from Figure 2. Curve-fits for (*D*, *E*, *F*) determined by Equation S1*and*S2. *G*, K_D_s for BuGZ and BuGZ mutants shown in μM with 95% confidence intervals. *H*, cartoon schematic of BuGZ oligomer (*left*) or BuGZ-NTD monomer (*right*) binding to bead-bound tubulin. *I*, cartoon schematic of unrestricted binding in solution of BuGZ (*left*) or BuGZ-NTD (*right*) to tubulin.

To further investigate BuGZ's ability to recognize tubulin nucleotide state, we used AlphaFold to predict BuGZ's protein structure (Fig. 3*A*) (43). Since most of the protein following BuGZ's N-terminal zinc finger motifs is predicted to be intrinsically disordered, the structure prediction beyond the N-terminal region is considered low confidence by AlphaFold (Fig. 3*A*). Next, we used AlphaFold to predict the structure of a BuGZ in-complex with a tubulin dimer (Fig. 3*B*). AlphaFold correctly predicted the tubulin dimer structure as previously determined by cryo-electron microscopy (44). Consistent with our previously reported finding, AlphaFold predicted an interaction between the N-terminus of BuGZ and the tubulin dimer (27, 29). AlphaFold also predicted that the C-terminal GLEBS motif of BuGZ, containing a short α-helix, is positioned close to the interface of α-tubulin and β-tubulin, implying that the GLEBS domain could interact with tubulin (Fig. 3*B*). Based on these analyses, we constructed and purified two BuGZ mutants: BuGZ-ΔGLEBS, in which the 32 amino acids of the GLEBS motif in the proline-rich region 2 (PRR2) are deleted, and BuGZ-NTD, the N-terminal 92 amino acids of BuGZ, which contain the two zinc fingers (Figs. 3*C*, S2, *C* and *D*).

Using the same binding assay described above, we measured the K_D_ for the binding of each BuGZ mutant to GTP-tubulin and GDP-tubulin. We found that BuGZ-ΔGLEBS binds to GTP-tubulin and GDP-tubulin with a K_D_ of 408.1 nM (95% CI: 156.3 nM–834.4 nM) and 54.2 nM (95% CI: 28.6 nM–90.6 nM), respectively (Figs. 3*D*, S4, *A* and *B*). We conclude that the GLEBS motif is not involved in BuGZ's binding to tubulin as these affinities are nearly identical to those measured for wild-type BuGZ. BuGZ-NTD also binds more weakly to GTP-tubulin than to GDP-tubulin. Its K_D_ for GTP-tubulin is 7.14 μM (95% CI: 5.53 μM–9.17 μM), while it binds GDP-tubulin with a tighter affinity of 1.47 μM (95% CI: 1.18 μM–1.82 μM) (Figs. 3*F*, S5, *A* and *B*). Thus BuGZ's N-terminal 92 amino acids containing its two zinc fingers, previously shown to be the BuGZ region responsible for microtubule binding (29), is sufficient for preferential binding to GDP-tubulin over GTP-tubulin, although it is not sufficient for maximum affinity binding.

The lower affinity of BuGZ-NTD for tubulin relative to full-length BuGZ indicates that BuGZ's intrinsically disordered C-terminal region, which drives phase separation (27), may play a role in BuGZ-tubulin interactions. To investigate this further, we performed binding assays with a BuGZ mutant that greatly reduces phase separation, BuGZ-13S (27). BuGZ-13S has 13 aromatic residues (phenylalanines and tyrosines) in the C-terminal region mutated to serines, but its C-terminus is otherwise intact. We found that the K_D_ for BuGZ-13S binding to GTP-tubulin and GDP-tubulin are 2.75 μM (95% CI: 1.97 μM–3.78 μM) and 710 nM (95% CI: 533 nM–926 nM), respectively (Figs. 3*E*, S2*C*, S6, *A* and *B*), tighter affinities than we measured for BuGZ-NTD but still weaker than wild-type BuGZ (Fig. 3G). The binding affinities we measured between GDP-tubulin or GTP-tubulin and various BuGZ constructs are depicted graphically in Figure 3G, and in a table in Figure S7. We interpret these results to mean that, while BuGZ's N-terminal domain is sufficient for nucleotide state-specific tubulin binding, its intrinsically disordered C-terminal region also contributes to tubulin binding, and optimal phase separation is required for maximum affinity.

Proteins that form condensates can form small oligomers even under conditions that disfavor phase separation (45, 46). Although our quantitative binding measurements were performed at 4 °C, which suppresses the formation of BuGZ condensates visible by light microscopy (27), small BuGZ oligomers may still form. These oligomers would lead to increased depletion of BuGZ by bead-bound biotinylated tubulin in our assay, which would manifest as higher measured affinities (Fig. 3*H*). In solution, we suspect that any extent of BuGZ phase separation would have the effect of concentrating both BuGZ and tubulin (27), effectively enhancing interactions (Fig. 3*I*).

### BuGZ promotes GTP exchange into GDP-tubulin

Nucleotide dissociation from tubulin is rapid (6). Since cells have a 10-fold higher concentration of GTP over GDP, it is thought that most GDP-tubulin produced when microtubules disassemble is rapidly converted to GTP-tubulin (23). However, different cellular states can greatly increase microtubule dynamics. For example, in mitosis, microtubule turnover is 18-fold faster than in interphase (24). Tubulin may require a nucleotide exchange factor during mitosis to sustain higher levels of microtubule dynamicity, as small perturbations can cause defects in the spindle, which can compromise timely and orderly cell division (24, 47). The 10-fold binding affinity bias of BuGZ to GDP-tubulin over GTP-tubulin prompted us to ask whether BuGZ could promote the exchange of GDP for GTP on tubulin.

**Figure 4:**
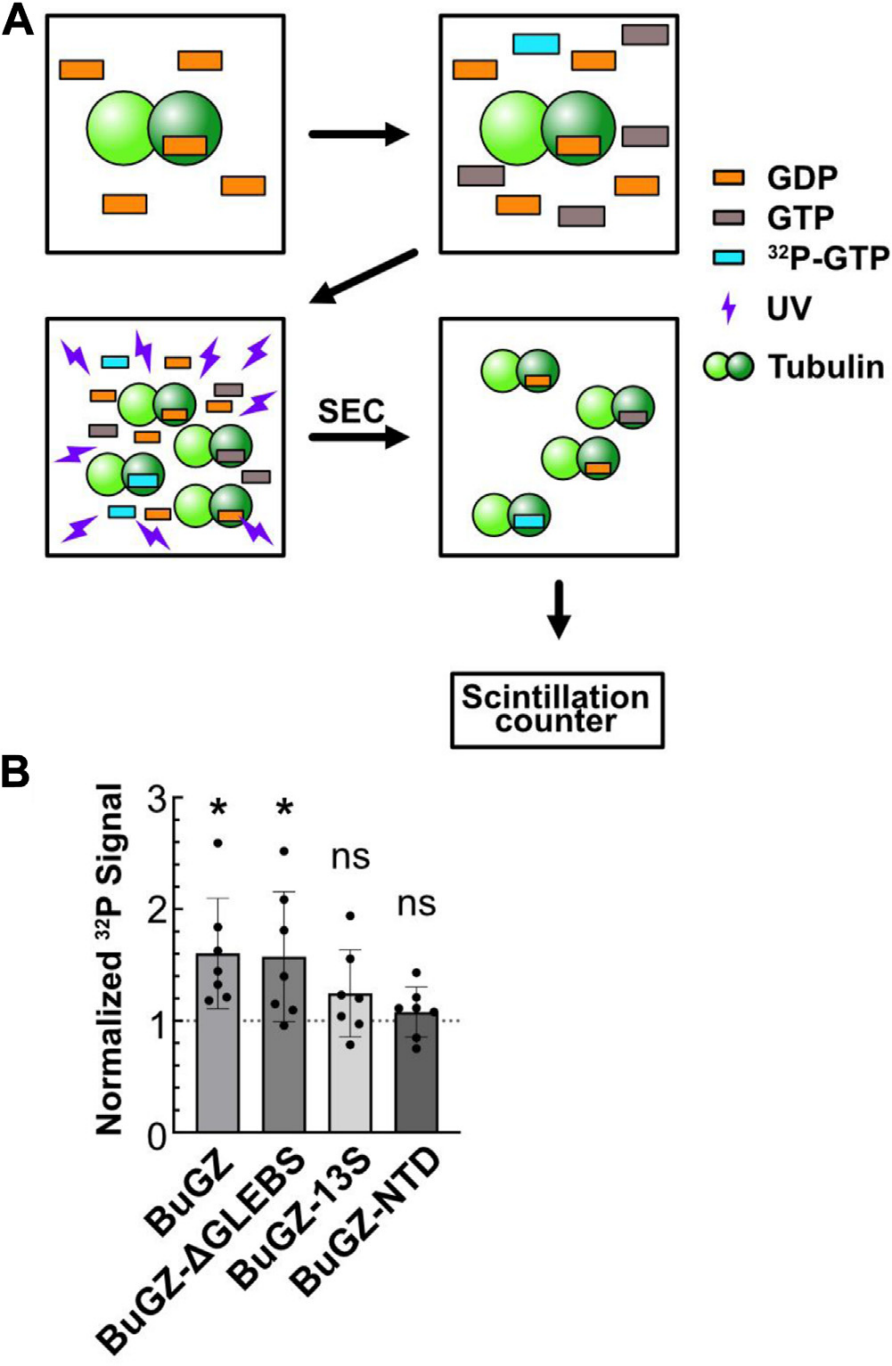
**BuGZ acts as a GEF for tubulin**. *A*, BuGZ GEF assay workflow. *B*, effect on GTP incorporation of tubulin in the presence of BuGZ measured by GTP α-^32^P signal. Mean and standard deviation shown. Wilcoxon signed-rank test: ∗*p* ≤ 0.05, ns, not significant, theoretical value = 1.

Tubulin's fast nucleotide dissociation *in vitro* (t_1/2_ ≅ 5 s) means that measuring a potential acceleration in exchange kinetics directly may be difficult (6). To overcome this challenge, we created experimental conditions that would reveal whether BuGZ promotes nucleotide exchange on tubulin at equilibrium. We measured the equilibrium levels of GTP α-^32^P incorporation into GDP-tubulin in the presence of BuGZ at room temperature, a condition in which BuGZ would undergo increased phase separation relative to our binding assays performed at 4 °C. The increased temperature would better replicate the physiological temperature for BuGZ's enzymatic activities as a GEF. 1 mM GTP containing 3 nM GTP α-^32^P was added to a solution of 100 μM GDP-tubulin and 1 mM GDP to achieve a 1:1 ratio of GTP:GDP. Then, BuGZ or BSA was added to 1.19 μM. We hypothesized that if BuGZ preferentially promotes nucleotide exchange on GDP-tubulin because of its stronger binding affinity, the presence of BuGZ would result in more GTP incorporated into tubulin at equilibrium in the presence of a 1:1 ratio of free GTP and GDP, which we could detect as an increase in tubulin bound to GTP α-^32^P. At equilibrium, nucleotides were cross-linked to tubulin *via* ultraviolet radiation and unbound nucleotides were removed by size-exclusion chromatography. The eluate containing tubulin and BuGZ was assessed by scintillation counting (Fig. 4*A*). In the presence of BuGZ, GTP incorporation increased by 60.1% (*p* = 0.01) relative to the control condition (Fig. 4*B*, Table S1). This shows that BuGZ acts as a tubulin GEF to promote GTP incorporation into tubulin.

Next, we tested our BuGZ mutants for nucleotide exchange activity using the same assay. At equilibrium, BuGZ-ΔGLEBS showed a significant increase of 57.4% (*p* = 0.03) in GTP incorporation relative to controls (Fig. 4*B*). Thus, just as BuGZ-ΔGLEBS binds tubulin similarly to wild-type BuGZ (Fig. 3*G*), it also promotes nucleotide exchange similar to wild-type BuGZ. Conversely, BuGZ-13S and BuGZ-NTD, which undergo diminished phase separation and which bind tubulin more weakly than wild-type BuGZ (Fig. 3*G*), showed statistically insignificant increases of GTP incorporation (Fig. 4*B*). However, we cannot rule out the possibility that these BuGZ constructs have GEF activity that our equilibrium assay does not have the sensitivity to detect. Thus, in our assay, BuGZ constructs that robustly undergo phase separation and tightly bind tubulin promote the exchange of GDP for GTP on tubulin.

## Discussion

Microtubule-associated proteins (MAPs) often bind preferentially to microtubule segments containing either GTP-tubulin (*e.g.*, at polymerizing plus-ends) or GDP-tubulin (*e.g.*, in the microtubule lattice) (48). However, nucleotide-specific interactions with tubulin have only been reported for 3 MAPs: CLIP-170, XMAP215, and stathmin. CLIP-170 binds to GDP- and GTP-tubulin with K_D_s of 55 nM and 33 nM, respectively, XMAP215 binds to GDP- and GTP-tubulin at K_D_ = 280 nM and K_D_ = 190 nM, respectively, and stathmin binds to GDP- and GTP-tubulin at K_D_ = 200 nM and K_D_ = 1 μM, respectively (39, 49, 50). These affinities contrast with BuGZ's 10-fold tighter binding to GDP-tubulin over GTP-tubulin (Figs. 2, *A* and *B*, 3*G*). Our finding should motivate more quantitative studies of various MAPs and their nucleotide-dependent interactions with tubulin (51, 52), which may uncover new mechanisms by which MAPs modulate microtubule assembly and function in different cellular contexts.

We found that BuGZ's N-terminal domain (BuGZ-NTD), which is both necessary and sufficient for binding tubulin (27, 29), binds preferentially to GDP-tubulin, although its affinity for both GDP- and GTP-tubulin is lower than full-length BuGZ (Fig. 3*F*). However, the same BuGZ construct does not promote significant tubulin nucleotide exchange (Fig. 4*B*). Our interpretation is that phase separation of BuGZ, which is severely diminished with BuGZ-NTD, may concentrate both tubulin and BuGZ in condensates, promoting more extensive BuGZ-tubulin interactions and facilitating nucleotide exchange to a level detectable by our equilibrium assay.

Our finding that BuGZ is a tubulin GEF is unexpected because of the prevailing idea that there is no need for one in the cell. The abundance of intracellular GTP, a high ratio of GTP:GDP in cells, and rapid nucleotide dissociation from tubulin are thought to quickly convert GDP-tubulin into GTP-tubulin (6, 23). However, some cellular processes are known to be rate-limited by diffusion and energy availability. For example, nucleocytoplasmic transport rates can increase or decrease when ATP or GTP availability is modulated (53). Microtubule growth is diffusion-limited such that concentrating microtubule plus ends causes slower polymerization due to a local depletion of free GTP-tubulin, whereas a local increase in free tubulin causes polymerization of nearby, but not distal, microtubules (26, 54, 55). In mitosis, rapid microtubule dynamics in the spindle could locally deplete free GTP-tubulin while increasing free GDP-tubulin. By localizing to the spindle and kinetochores (28, 29), and binding GDP-tubulin from microtubule depolymerization, BuGZ could increase GTP-tubulin production. Following nucleotide exchange, the 10-fold lower affinity for GTP-tubulin would mediate BuGZ's release. How BuGZ functions as a GEF remains unclear, but its preferential binding to GDP-tubulin suggests that it may not facilitate equal nucleotide exchange of GDP- and GTP-tubulin as seen for other GEFs but instead specifically facilitate nucleotide exchange of GDP-tubulin. Since the zinc finger domain of BuGZ can be produced in bacteria in high quantity and purity, it should be possible to solve the structure of the BuGZ-tubulin complex, which should shed light on how BuGZ functions as a tubulin GEF.

## Experimental procedures

### Cloning, protein expression, and protein purification

Methods for *X. laevis* BuGZ construct design, expression, and purification are described in Jiang *et al.*, *Cell*, 2015 (27) with further detail provided in Supporting Information. Representative images of the purified protein preparations used in this study, resolved by SDS-PAGE and stained with Coomassie, are shown in Figure S2, *A*–*D*.

### Measurement of BuGZ binding to taxol-stabilized microtubules, GTP-tubulin, and GDP-tubulin

Protocol for quantitative binding assays *via* supernatant depletion is described in Pollard, *Mol. Biol. Cell*, 2010 and Lee, *et al.*, *J. Biol. Chem*, 1999 (56, 57) with further detail provided in Supporting Information.

### Gel electrophoresis and immunoblot analysis

Gel electrophoresis and immunoblots were performed using standard methods. Antibodies: Anti-6X His tag antibody (Abcam ab18184) 1:1000. LI-COR IRDye 680RD Goat anti-Mouse IgG (926–68070) 1:10,000. Further details are provided in Supporting Information.

### Nucleotide exchange assay

Methodology for measuring tubulin nucleotide state is previously described in Oegema *et al.*, *J. Cell Biol.*, 1999 (58) with further detail provided in Supporting Information.

### Data analysis

Statistical analyses were performed as described in Pollard, *Mol. Biol. Cell*, 2010 (56) with further detail provided in Supporting Information.

### Alphafold protein structure prediction

Protein structure predictions of BuGZ (UniProt: Q7ZXV8), α-tubulin (Uniprot: Q71U36), and β-tubulin (Uniprot: Q9H4B7) were generated AlphaFold v2.3.2. Predicted structures were color-coded using ChimeraX.

## Data availability

All data described in this study are contained in the manuscript.

## Supporting information

This article contains supporting information.

## Conflict of interest

The authors declare that they have no conflicts of interest with the contents of this article.
