# Supplementary material for: A tubulin-binding protein that preferentially binds to GDP-tubulin and promotes GTP exchange
Source: J Biol Chem. 2025 Jun 19;301(8):110401. doi: 10.1016/j.jbc.2025.110401 (PMC12302650; doi:10.1016/j.jbc.2025.110401)
Supplement: Supplementary Data [file mmc1.docx]

### A tubulin-binding protein that preferentially binds to GDP-tubulin and promotes GTP exchange

Wesley J. Yon^1,2^, Taekjip Ha^2,3,4,5^, Yixian Zheng^1,2,✝^, and Ross T.A. Pedersen^1^

^1^ Department of Embryology, Carnegie Institution for Science, Baltimore, MD, USA

^2^ Cell, Molecular, Developmental Biology, and Biophysics Program, Johns Hopkins University, Baltimore, MD, USA

^3^ Department of Pediatrics, Harvard Medical School, Boston, MA, USA

^4^ Program in Cellular and Molecular Medicine, Boston Children’s Hospital, Boston, MA, USA

^5^ Howard Hughes Medical Institute, Boston, MA, USA

^✝^Corresponding author: [zheng@carnegiescience.edu](mailto:zheng@carnegiescience.edu)

### Supporting Information

**Cloning, protein expression, and protein purification**

*X. laevis* BuGZ cDNA was cloned into Gibco pFastBac Dual Expression Vector via Gibson Assembly. In this plasmid, BuGZ (NCBI: NM_001086855.1) is tagged on its amino terminus with: four leader amino acids, 6x His tag, GS linker, and TEV protease cleavage site (full tag sequence: MSYY-HHHHHH-GSG_4_SG_4_S-ENLYFQG). Sf9 cells (Gibco) were transfected using Gibco Cellfectin II Reagent according to manufacturer’s instructions to generate P0 viral stock. After 3 rounds of viral amplification, the resulting P3 virus was used to infect Sf9 cells for protein expression. 72 hrs post-infection, cells were collected via centrifugation at 1,000 x g, snap-frozen in liquid nitrogen, and stored at -80 °C. For purification, pellets were resuspended in lysis buffer (LyB): 20 mM KH_2_PO_4_, 500 mM NaCl, 25 mM Imidazole, 1 mM MgCl_2_, 1 mM 2-mercaptoethanol, 2.5% Glycerol, 0.01% Triton X-100, Roche cOmplete Protease Inhibitor Cocktail, pH 7.4. The cell suspension was sonicated using a Misonix Sonicator 3000 (2 min total process time, 30 s on, 30 s off, power level 2.0) on ice and clarified by centrifugation at 15,000 x g for 30 min. Clarified lysate was run through a 0.22 μm filter (Millipore Sigma SLGPR33RB). Lysate was loaded onto a 1 mL HisTrap HP column (Cytiva) equilibrated in Buffer A (LyB without protease inhibitor cocktail) and ran on a Cytiva Äkta Pure FPLC. The column was then washed with 1x lysate volume of 85% Buffer A/15% Buffer B (20 mM KH_2_PO_4_, 150 mM NaCl, 300 mM Imidazole, 1 mM MgCl_2_, 1 mM 2-mercaptoethanol, 2.5% Glycerol, 0.01% Triton X-100, pH 7.4). Bound protein was eluted with a 30 mL linear gradient from 15% to 100% Buffer B. BuGZ-containing fractions were identified via SDS-PAGE and staining with Invitrogen SimplyBlue SafeStain. BuGZ-positive fractions were pooled and concentrated to < 500 μL using Millipore Amicon Ultra centrifugation units with 30 kDa MW cut off (10 kDa MW cut-off for BuGZ-NTD). Contaminating proteins were further removed from the concentrated BuGZ solution by gel filtration using a Superdex 200 Increase 10/300 GL column equilibrated in Buffer C (80 mM PIPES pH 6.8, 100 mM KCl, 1 mM MgCl_2_, 50 mM Sucrose, 1 mM EGTA). Fractions of the eluate containing BuGZ were identified via SDS-PAGE, pooled, concentrated using Millipore Amicon Ultra centrifugation units, and snap-frozen in liquid nitrogen for storage at -80 °C. The purity and concentration of BuGZ was determined by comparing in-gel SimplyBlue SafeStain staining to staining of known amounts of bovine serum albumin.

**Measurement of BuGZ binding to Taxol-stabilized microtubules, GTP-tubulin, and GDP-tubulin**

Microtubule binding: Tubulin (Cytoskeleton T240) and biotin-tubulin (Cytoskeleton T333P) were mixed at a 10:1 ratio in BRB80 (80mM PIPES, 1mM MgCl_2_, 1mM EGTA, pH 6.8) + 1 mM GTP. The tubulin solution was diluted 1:1 in BRB80, 2 mM DTT, 2 mM GTP, 20 μM taxol (taxol buffer 1) and incubated for 20 min at 37 °C. Pierce Streptavidin Magnetic Beads were equilibrated in BRB80, 1 mM DTT, 1 mM GTP, 10 μM taxol (taxol buffer 2). The microtubule assembly mixture was added to streptavidin-coated beads and incubated for 20 min at 4 °C, then washed 3 times with taxol buffer 2. The beads were resuspended in a 10 μL solution containing taxol buffer 2 and 1.19 μM BuGZ. After 20 min incubation at 4 °C, the beads were collected with a magnet, and the supernatant was removed for immunoblot analysis. Taxol-stabilized microtubules were passed through a 30 G syringe needle 10 times before addition to streptavidin-coated beads for sheared microtubule experiments.

Tubulin binding: Biotin-tubulin, 200 μM nocodazole, 1 mM GTP or GDP, and BRB80 were added to Pierce Streptavidin Magnetic Beads to decorate them with the desired amount of tubulin. After 20 min incubation at 4 °C, beads were washed 3 times in the same buffer and the supernatant was removed. The magnetic beads, now decorated with biotin-tubulin, were resuspended in equal volumes of a solution containing 1.19 μM BuGZ, 200 μM nocodazole, 1 mM GTP or GDP, and BRB80. After 15 min incubation at 4 °C, beads were collected with a magnet and supernatant was retained for immunoblot analysis.

**Gel electrophoresis and immunoblot analysis**

Supernatants from binding experiments were boiled at 95 °C for 10 min and spun in a centrifuge at 10,000 x g for 3 min. SDS-PAGE was performed in a Tris-Glycine buffer (25 mM Tris, 250 mM glycine, 3.5 mM SDS). Following electrophoresis, proteins were transferred onto nitrocellulose membrane (Cytiva Amersham 10600002) at 35 V for 2 hrs at 4 °C in transfer buffer (50 mM Tris, 125 mM glycine, 3.5 mM SDS, 20% methanol). After transfer, membranes were incubated in a block buffer (5% w/v skim milk, Tris-buffered saline (TBS) pH 7.4) for 1 hr at room temperature, then probed with primary antibodies to the 6x His tag (for BuGZ recognition) in block buffer + 0.02% Tween-20, for 1 hr at room temperature. After 3 washes with TBS, membranes were incubated in a secondary antibody solution (TBS, 0.02% Tween-20, antibody) for 1 hr at room temperature. After 3 washes in TBS, membranes were imaged using a LI-COR CLx system and quantified by densitometry using the associated ImageStudio software. In our hands, our primary antibody against 6x His tag (Abcam ab18184) recognized a band of the appropriate size for each purified 6x His-tagged protein.

**Nucleotide exchange assay**

A solution containing 100 μM tubulin, 1 mM GDP, BRB80 was mixed with a 1/10 volume of 10X nucleotide exchange buffer (10 mM GTP, 33.3 nM GTP α-^32^P (PerkinElmer BLU506H250UC), 1 mM nocodazole, BRB80) for a final solution containing 91 μM tubulin, 0.9 μM GDP, 0.9 μM GTP, 2.7 nM GTP α-^32^P, 91 μM nocodazole, and BRB80. Immediately, a BRB80 solution containing either BuGZ or BSA was added to a final concentration of 1.19 μM of the protein. Corresponding controls were produced in the same manner except without the addition of tubulin. Mixtures were incubated at room temperature for 15 min. Then, samples were treated with UV for 5 min in a Stratagene UV Stratalinker 1800. Free nucleotides were removed with size-exclusion columns (BioRad 7326222) equilibrated to BRB80 buffer according to manufacturer’s instructions. Flowthrough was mixed with a scintillation cocktail (RPI Bio-Safe II) and GTP α-^32^P was measured using PerkinElmer Tri-Carb 2810 TR. The measured signal from the (-) tubulin conditions for BSA or BuGZ were subtracted as background from its corresponding tubulin + BuGZ or BSA readouts. Then, the data were normalized as a ratio of the BuGZ to BSA ^32^P signals.

**Data analysis**

Statistical analyses were performed using Graphpad Prism 10. Equilibrium dissociation constants were determined for conditions where the K_D_ was near or far exceeding the total concentration of BuGZ (BuGZ to taxol-MT, BuGZ to GTP-tubulin, BuGZ-ΔGLEBS to GTP-tubulin, BuGZ-13S to GDP- and GTP-tubulin, BuGZ-NTD to GDP- and GTP-tubulin), using the function:


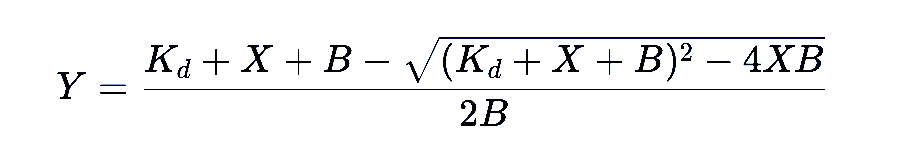
(Equation S1)

where X is the microtubule/tubulin concentration, B is the concentration of BuGZ, and Y

is the BuGZ fraction bound.

For conditions where the K_D_ was far less than the total concentration of BuGZ (BuGZ to GDP-tubulin, BuGZ-ΔGLEBS to GDP-tubulin), equilibrium dissociation constants were determined using Prism 10’s ‘Hyperbola’ function:


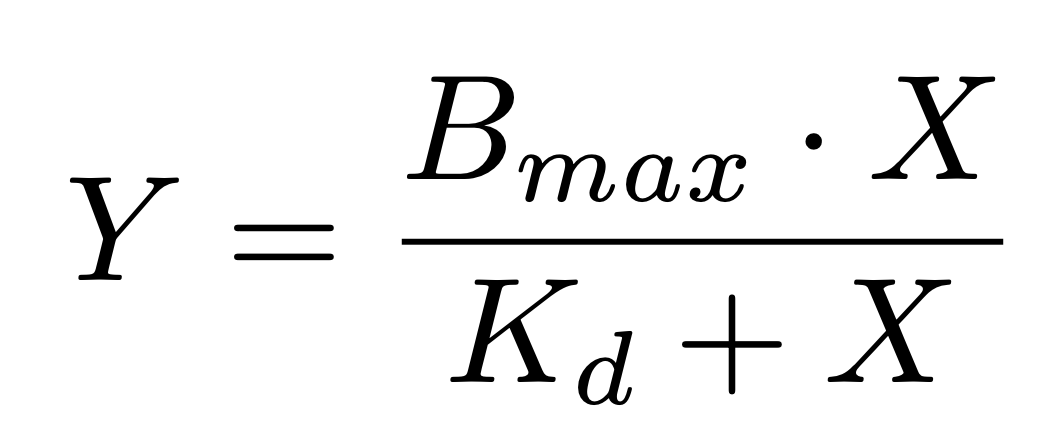
(Equation S2)

where B_max_ = 1, X is the concentration of BuGZ, and Y is the BuGZ fraction bound. Nucleotide exchange assays were analyzed using Wilcoxon signed-rank test against a hypothetical value of 1.

**Figure S1**

**
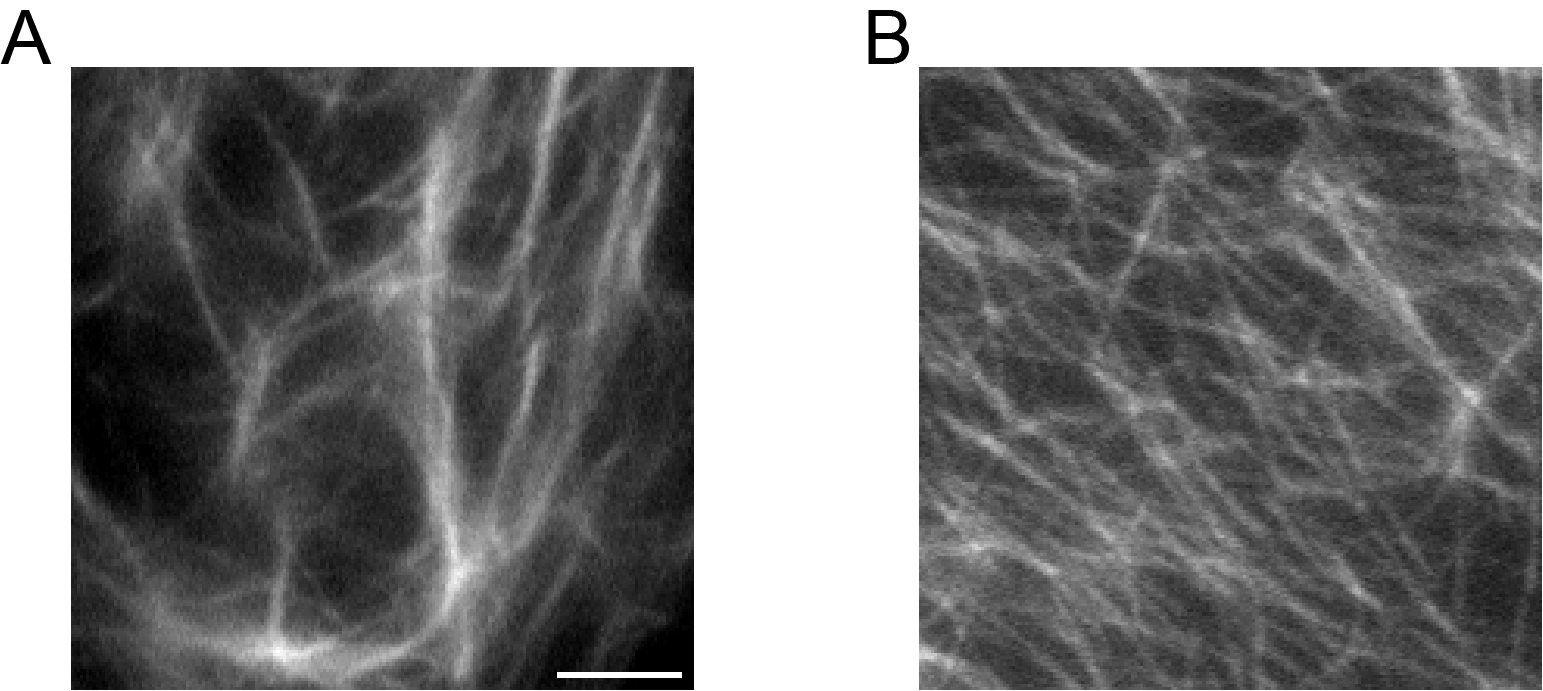
**

**Figure S1** Taxol-stabilized microtubules persist at 4° C. (A) Microtubules polymerized in the presence of taxol at 37° C. (B) Taxol-stabilized microtubules after 30 min. 4° C incubation. Scale bar, 5 µM.

**Figure S2**

**
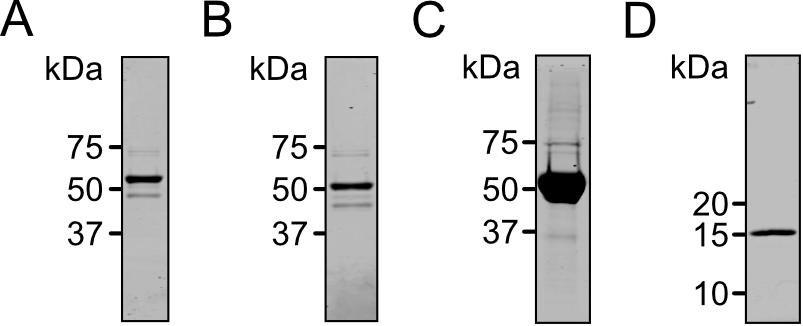
**

**Figure S2** BuGZ and BuGZ mutant protein purification results. (A) BuGZ. (B) BuGZ-ΔGLEBS. (C) BuGZ-13S. (D) BuGZ-NTD. Protein ladder molecular weight markers indicated on the left for each sample.

**Figure S3**


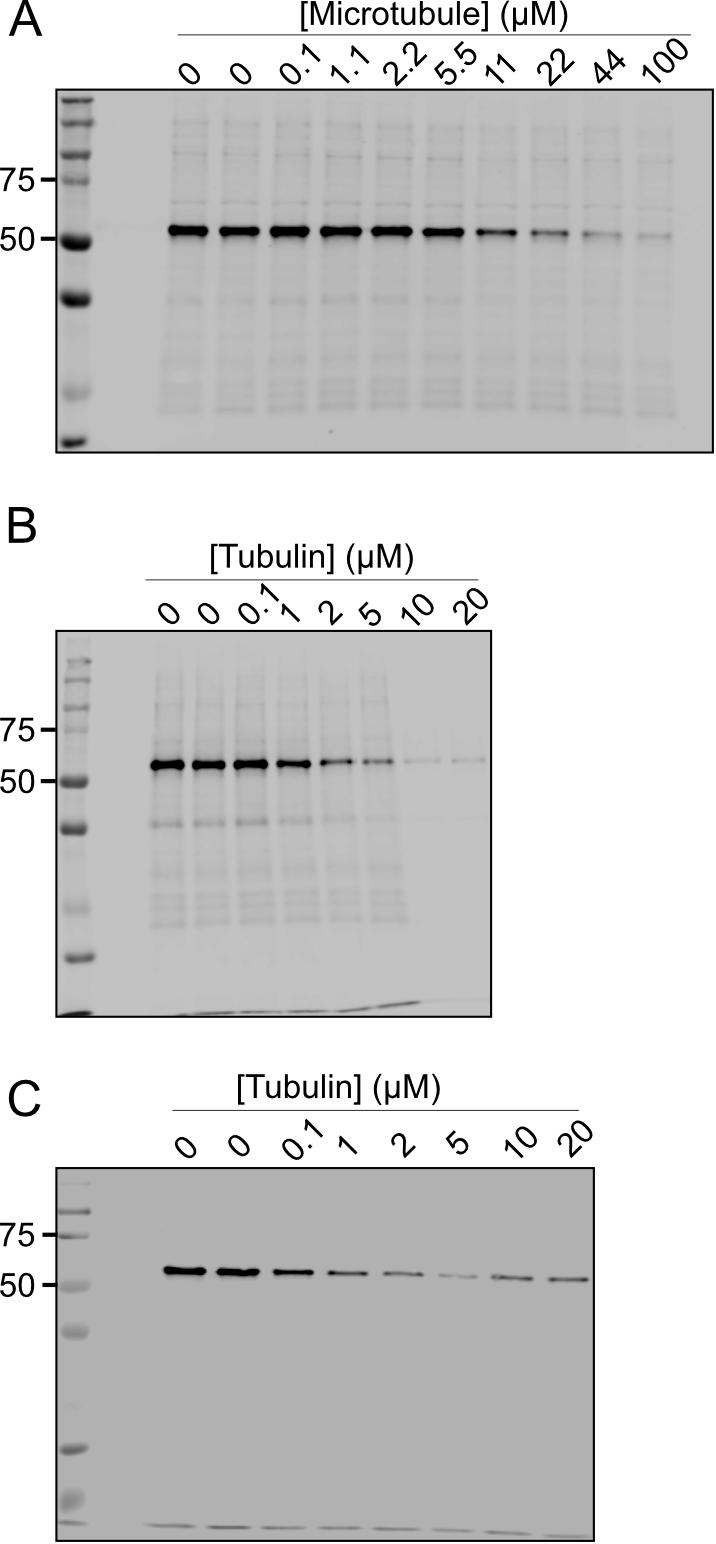


**Figure S3** BuGZ binding assay representative immunoblots (A) Taxol-stabilized microtubules. 0.1-2.2 µM conditions not used for final analysis. (B) GTP-tubulin. (C) GDP-tubulin. Protein ladder molecular weight markers indicated on the left for each sample.

**Figure S4**


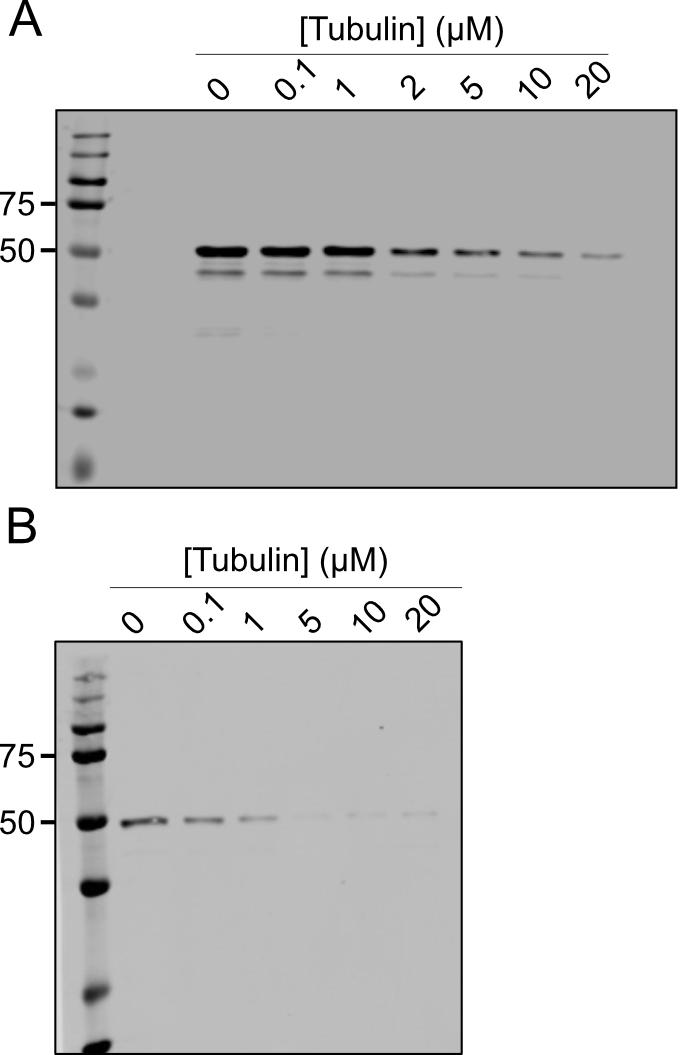


**Figure S4** BuGZ-ΔGLEBS binding assay representative immunoblots (A) GTP-tubulin. (B) GDP-tubulin. Protein ladder molecular weight markers indicated on the left for each sample.

**Fig S5**

**
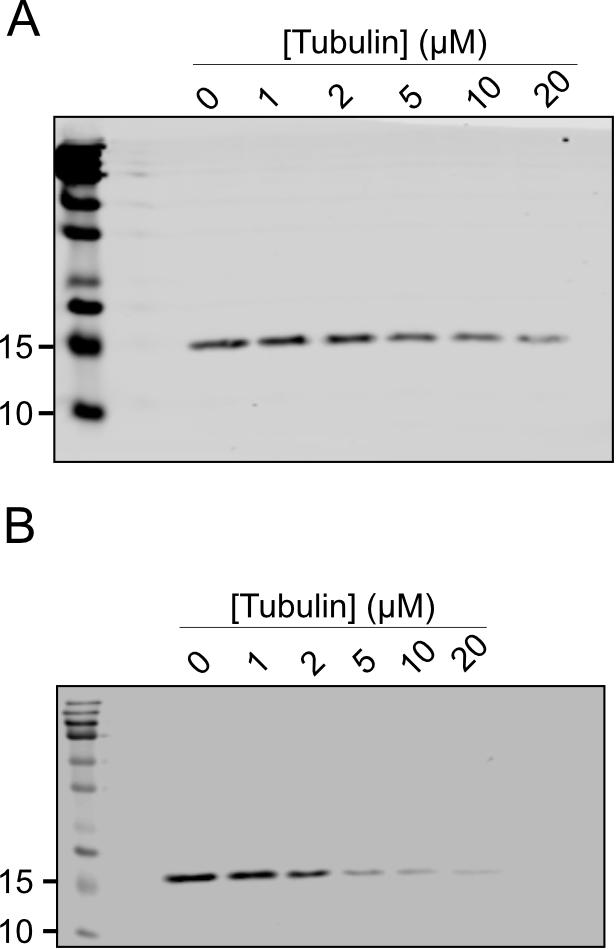
**

**Figure S5** BuGZ-NTD binding assay representative immunoblots (A) GTP-tubulin. (B) GDP-tubulin. Protein ladder molecular weight markers indicated on the left for each sample.

**Figure S6**

**
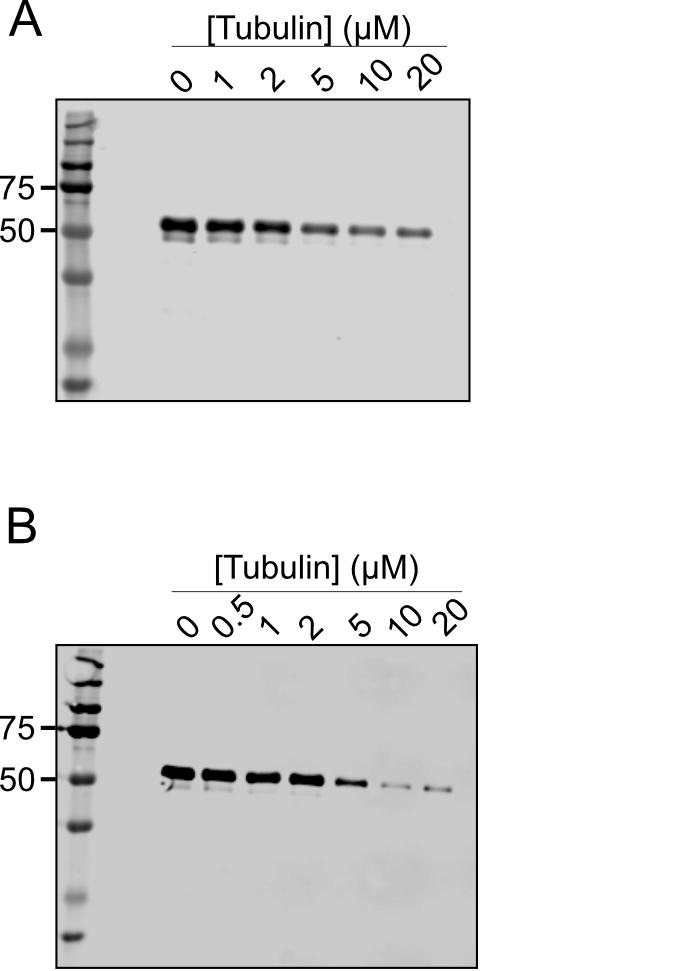
**

**Figure S6** BuGZ-13S binding assay representative immunoblots (A) GTP-tubulin. (B) GDP-tubulin. Protein ladder molecular weight markers indicated on the left for each sample.

**Figure S7**

**
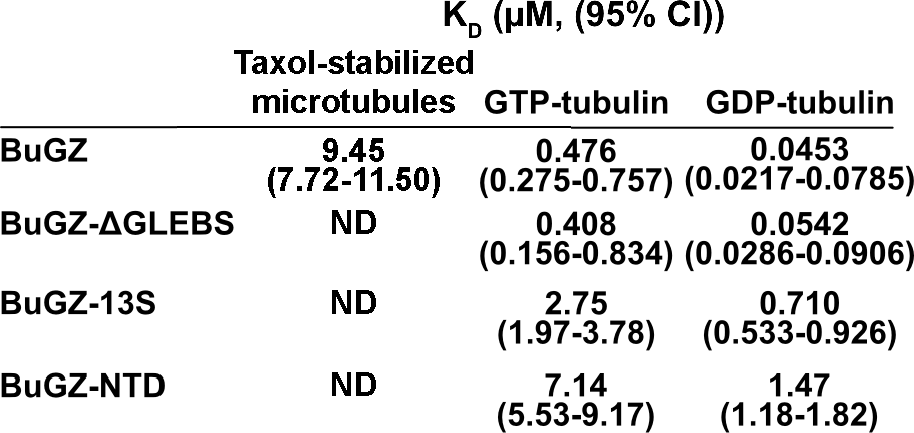
**

**Figure S7** Table of equilibrium dissociation constants measured in this study. ND: not determined.
